# Whole‐blood dysregulation of actin‐cytoskeleton pathway in adult spinal muscular atrophy patients

**DOI:** 10.1002/acn3.51092

**Published:** 2020-06-17

**Authors:** Jennifer J. Siranosian, Flavia C. Nery, Christiano R. R. Alves, Benjamin A. Siranosian, Nicholas J. Lyons, Eric J. Eichelberger, Reid Garner, Salomé Da Silva Duarte Lepez, Alec J. Johnstone, Aravind Subramanian, Kathryn J. Swoboda

**Affiliations:** ^1^ Department of Neurology Center for Genomic Medicine Massachusetts General Hospital Boston MA USA; ^2^ Broad Institute of MIT and Harvard Cambridge MA USA

## Abstract

**Objective:**

Recent advances in therapeutics have improved prognosis for severely affected spinal muscular atrophy (SMA) type 1 and 2 patients, while the best method of treatment for SMA type 3 patients with later onset of disease is unknown. To better characterize the SMA type 3 population and provide potential therapeutic targets, we aimed to understand gene expression differences in whole blood of SMA type 3 patients (n = 31) and age‐ and gender‐matched controls (n = 34).

**Methods:**

We performed the first large‐scale whole blood transcriptomic screen with L1000, a rapid, high‐throughput gene expression profiling technology that uses 978 landmark genes to capture a representation of the transcriptome and predict expression of 9196 additional genes.

**Results:**

The primary downregulated KEGG pathway in adult SMA type 3 patients was "Regulation of Actin Cytoskeleton,” and downregulated expression of key genes in this pathway, including *ROCK1*, *RHOA*, and *ACTB,* was confirmed in the same whole blood samples using RT‐qPCR. SMA type 3 patient‐derived fibroblasts had lower expression of these genes compared to control fibroblasts from unaffected first‐degree relatives. Overexpression of SMN levels using an AAV vector in fibroblasts did not normalize *ROCK1*, *RHOA*, and *ACTB* mRNA expression, indicating the involvement of additional genes in cytoskeleton dynamic regulation.

**Interpretation:**

Our findings from whole blood and patient‐derived fibroblasts suggest SMA type 3 patients have decreased expression of actin cytoskeleton regulators. These observations provide new insights and potential therapeutic targets for SMA patients with longstanding denervation and secondary musculoskeletal pathophysiology.

## Introduction

Spinal muscular atrophy (SMA) is characterized by progressive motor neuron denervation resulting in chronic muscle weakness and atrophy caused by mutations in the *survival motor neuron 1* (*SMN1*) gene.^1^, ^2^ SMA is divided into three primary clinical subtypes based on age of onset and maximum achievement of key motor abilities. SMA type 1 and 2 patients have onset in infancy and never walk, while SMA type 3 patients have a more variable age of onset and a normal lifespan.^3^, ^4^, ^5^ Although SMA type 3 patients achieve the ability to walk independently, progressive muscular weakness in the absence of disease‐modifying therapy results in progressive loss of predominantly proximal muscle strength and functional mobility. Despite significant improvement in survival and motor function in SMA infants and children treated with recently FDA‐approved novel molecular and gene therapies,^1^, ^6^, ^7^ further studies are needed to determine the benefits and risks of these novel strategies in adult patients with chronic denervation, atrophy, and weakness. Pre‐existing immunity to gene therapy vectors, challenges in the delivery of current therapies, and lack of information regarding the need for treatment of peripheral tissues, including skeletal muscle, remain as barriers to determining the most ideal treatment approach in our adult SMA patients.^8^ Therefore, identifying novel therapeutic targets to improve functional outcomes in older children and adult SMA patients with chronic disease remains a priority for researchers and clinicians.^8^

Whole blood transcriptome data in adult SMA patients have not been previously studied. Here, we applied the L1000 profiling technology and Gene Set Enrichment Analysis (GSEA) to explore potential pathways affected in whole blood from SMA type 3 adult patients and age‐ and gender‐matched controls. L1000 is a rapid, high‐throughput gene expression profiling technology that utilizes the expression of 978 landmark gene transcripts to predict the expression of 9196 additional genes.^9^ The comparatively low cost of L1000 profiling and strong correlation with RNA‐seq expression profiles sets it apart from other techniques. Reagent cost is ~2 US dollars per sample, making its application feasible for exploratory projects involving a large number of samples. Since neither L1000 nor RNA‐seq directly measures the expression of most of the genes, quantitative RT‐PCR experiments were performed to validate our primary observations and explore other genes in indicated pathways.

Comparing expression profiles from adult SMA type 3 and healthy age‐ and gender‐matched control subjects, GSEA analysis identified "Regulation of Actin Cytoskeleton” as the primary downregulated *Kyoto Encyclopedia of Genes and Genomes* (KEGG) pathway in SMA type 3 patients. RT‐qPCR and fibroblast studies then confirmed these findings. The actin cytoskeleton is a collection of actin filaments and their accessory and regulatory proteins that play a critical role in regulating cell motility and contractility.^10^, ^11^ Actin cytoskeleton dynamics use metabolic energy to produce pushing, pulling, and resistance forces responsible for multiple motility functions,^10^, ^11^ such as the fast motility of immune cells which allows them to survey tissues to find and destroy pathogens.^10^ Impaired actin cytoskeleton dynamics has previously been demonstrated in motoneurons derived from SMA type 1 patients,^12^, ^13^ however, little is known about how the actin cytoskeleton is regulated in cells or tissues in SMA patients.

## Methods

### Study approval and subjects

Written informed consent was obtained from all subjects under Institutional Ethics Review Board at the University of Utah (protocol #8751) and Massachusetts General Hospital (protocol #2016P000469). Adult SMA type 3 patients (n = 31) and healthy age‐ and gender‐matched control subjects (n = 34) participated in this study. All blood samples were collected at a basal state and this study did not include patients receiving valproic acid, nusinersen, AVXS‐101, RO7034067, or any other therapy for SMA. *Survival motor neuron 2* (*SMN2*) copy number was determined for each SMA patient using qPCR.^14^, ^15^ Maximum ulnar compound muscle action potential (CMAP) was determined by recording from the abductor digiti minimi muscle following maximum ulnar nerve stimulation at the wrist from at least five separate G1 electrode placements, as previously described.^16^, ^17^ Table 1 presents the characteristics of both SMA and healthy control subjects.

**Table 1:** Demographic and clinical characteristics of SMA type 3 patients and healthy controls

	Healthy Controls (n = 31)	SMA Type 3 (n = 34)	*P‐value*
Age	37.1 ± 6.7	36.8 ± 8.1	0.87
Gender, no. (%)			
Male	16 (51.6)	17 (50.0)	
Female	15 (48.4)	17 (50.0)	
Race, no. (%)			
White	28 (90.3)	33 (97.1)	
Asian	2 (6.5)	1 (2.9)	
Black	1 (3.2)	0 (0)	
Ethnicity, no. (%)			
Not Hispanic	28 (90.3)	32 (94.1)	
Hispanic	3 (9.7)	1 (2.9)	
Unknown	0 (0)	1 (2.9)	
CMAP score	—	7.1 ± 3.4 (n = 31)	
*SMN2*, no. (%)			
1	—	1 (2.9)	
2	—	3 (8.8)	
3	—	10 (29.4)	
4+	—	19 (55.9)	
Unknown	—	1 (2.9)	

### Cell culture

Fibroblasts were derived from skin biopsies from two SMA patients with three *SMN2* copies and two unaffected related healthy control subjects with at least one *SMN1* copy. Fibroblasts were cultured in Dulbecco's modified Eagle's medium (DMEM) supplemented with 10% fetal bovine serum (DMEM; Gibco, NY). Transient SMN knockdown was performed in fibroblasts from healthy control subjects using SMN siRNA (3.5nM; Cat # 4392420; Thermo Fisher Scientific, MA) or scrambled siRNA (3.5nM; Cat # 4390843; Thermo Fisher Scientific, MA). Transfection was performed 2 days after cell seeding using a complex with siRNAs and Lipofectamine RNAiMAX Transfection Reagent (Thermo Fisher Scientific, MA) following the datasheet. RNA was extracted 2 days posttransfection. To replace SMN in fibroblasts from a SMA patient, cells were transduced with adeno‐associated virus (AAV) (MOI 100) to express human *SMN1* transcript (NCBI Reference Sequence: NM_000344.4; AAV‐223756; Vector Biolabs, Malvern, PA, USA) or GFP (Lot# 190527‐190627; Vector Biolabs, Malvern, PA, USA). Media were replaced 12 hours after transduction and changed every other day. RNA was extracted 4 days posttransduction.

### L1000 and GSEA

Total RNA was isolated from whole blood samples using a PAXgene Blood RNA Kit (Qiagen, Hilden, Germany). RNA samples were plated in a 384‐well plate and transcripts were captured on oligo‐dT primers then reverse transcribed. cDNA was subjected to ligation‐mediated amplification using probes carrying a gene‐specific sequence, an identifying barcode, and a 5’ biotin label. Polystyrene beads of distinct fluorescent colors were hybridized to each probe via complementary barcode sequence binding, and the results were stained. Fluorescent color and intensity were analyzed separately to determine gene identity and quantify expression abundance. The expression values of 978 transcripts were used to infer the remaining 9,196 genes as previously described.^9^ To calculate fold change, normalized expression values were processed using the R package *limma* ^18^ (code in supplement). Briefly, a linear model, specified by a design matrix accounting for test group and age block by decade, was fitted to the data matrix. Pair‐wise comparisons were made between SMA and healthy controls, and an empirical Bayes method was used to moderate the standard errors of the log‐fold change estimates.^18^ Genes with a *P*‐value < 0.05 were subjected to GSEA^19^, ^20^ to identify dysregulated pathways in SMA type 3 patients. We present the 10 top affected pathways ranked based on ‐log(*P*‐value).

### RT‐qPCR

RNA was isolated from whole blood using PAXgene Blood RNA Kit (Qiagen, Hilden, Germany) or from fibroblasts using RNeasy Plus Universal Kits (Qiagen, Hilden, Germany). RNA was reverse transcribed using the RT^2^ First Strand Kit (Qiagen, Hilden, Germany) protocol and cDNA was amplified using SYBR Green (Qiagen, Hilden, Germany) in a LightCycler 480 Instrument II (Roche, Basel, Switzerland). For each gene, mRNA expression was calculated relative to *TATA‐binding protein* (*TBP1*). Primer sequences are provided in Table 2.

**Table 2:** List of primer sequences used for RT‐qPCR

Gene	Forward (5′> 3′)	Reverse (5′> 3′)
*ACTB*	ACA‐GAG‐CCT‐CGC‐CTT‐TGC‐C	GAT‐ATC‐ATC‐ATC‐CAT‐GGT‐GAG‐CTG‐G
*ACTA1*	AAG‐ATC‐AAG‐ATC‐ATC‐GCC‐CCG	CCT‐CGT‐CGT‐ACT‐CCT‐GCT‐TG
*ACTA2*	GCC‐AAG‐CAC‐TGT‐CAG‐GAA‐TC	TTG‐TCA‐CAC‐ACC‐AAG‐GCA‐GT
*ACTC1*	ATG‐CCA‐TCA‐TGC‐GTC‐TGG‐AT	ACG‐TTC‐AGC‐AGT‐GGT‐GAC‐AA
*ACTG1*	GTT‐TCT‐CTG‐CCG‐GTC‐GCA‐AT	CCC‐GAC‐GAT‐GGA‐AGG‐AAA‐CA
*RHOA*	CGT‐TAG‐TCC‐ACG‐GTC‐TGG‐TC	CAG‐CCA‐TTG‐CTC‐AGG‐CAA‐C
*ROCK1*	AAG‐AGG‐GCA‐TTG‐TCA‐CAG‐CA	AGC‐ATC‐CAA‐TCC‐ATC‐CAG‐CA
SMN	ACA‐ACA‐GTG‐GAA‐AGT‐TGG‐GGA	TGA‐AGC‐AAT‐GGT‐AGC‐TGG‐GT
*TBP1*	GCA‐TCA‐CTG‐TTT‐CTT‐GGC‐GT	AGA‐GCA‐TCT‐CCA‐GCA‐CAC‐TC

### Statistical Analysis

Data are presented as mean ± SEM with filled symbols representing individual values. Statistical analyses were performed using GraphPad Prism 7 software (GraphPad Software, Inc.). Unpaired or paired Student’s *t* tests were used to compare groups. Statistical significance was defined as *P* < 0.05.

## Results

To establish a transcriptome profile for adult SMA type 3 patients, we performed the L1000 profiling technology using whole blood from 31 SMA type 3 patients and 34 healthy age‐ and gender‐matched control subjects (Table 1). As expected, most (>85%) of the SMA type 3 patients had three or more *SMN2* copies (Table 1). Ulnar CMAP amplitudes were typical of those previously reported for SMA type 3 patients (7.1 ± 3.4), indicating less severe denervation in the ulnar‐innervated abductor digiti minimi (ADM) muscle as compared to patients with SMA type 1 or type 2.^4^ Control subjects had at least one *SMN1* copy and had no signs of muscle weakness or atrophy.

**Figure 1:**
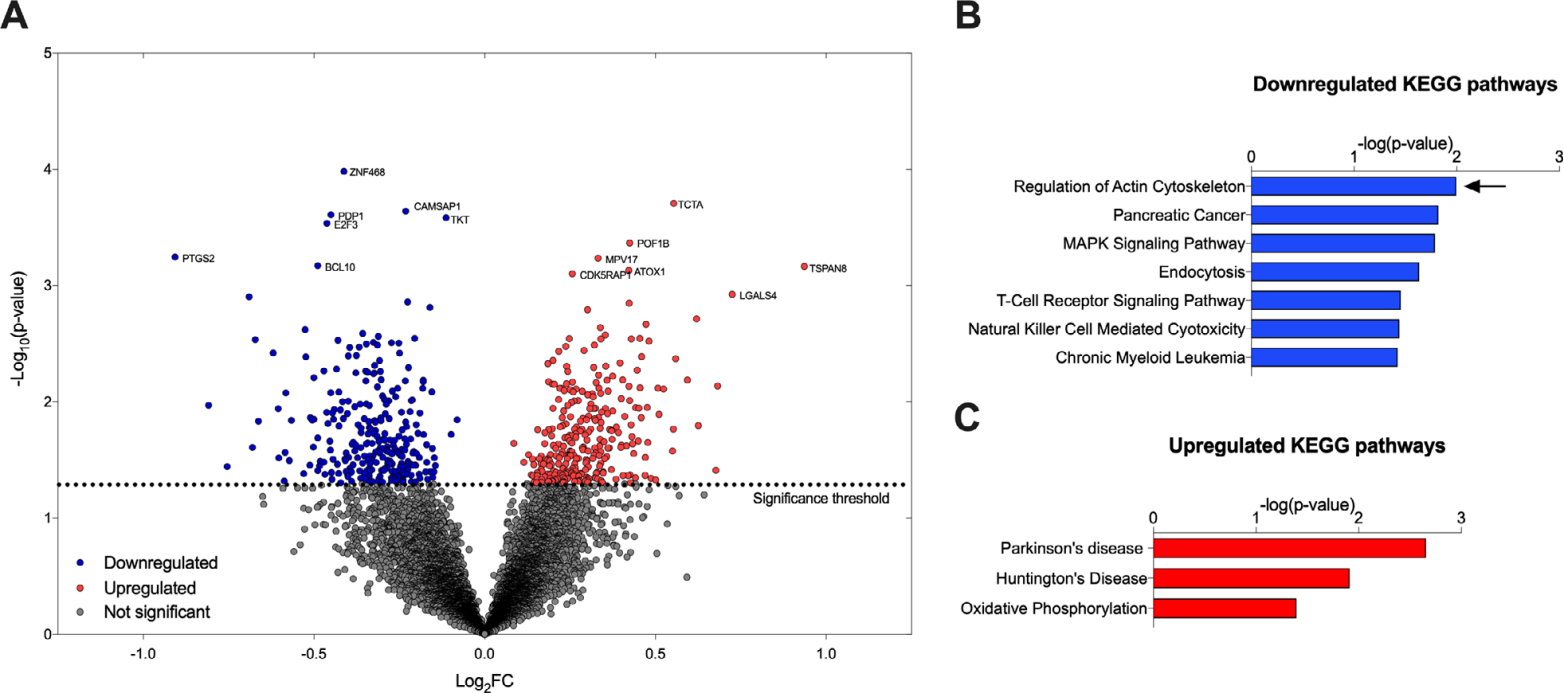
Transcriptomic analysis in SMA type 3 patients and healthy controls. (A) Volcano plot of 10,174 genes in L1000 experiment. Downregulated (blue) and upregulated (red) genes were selected for GSEA using a simple cutoff at *P* ≤ 0.05. GSEA reports (B) seven significantly downregulated and (C) three significantly upregulated KEGG pathways in SMA type 3 patients compared to healthy controls. Regulation of Actin Cytoskeleton was the most significantly downregulated pathway

**Figure 2:**
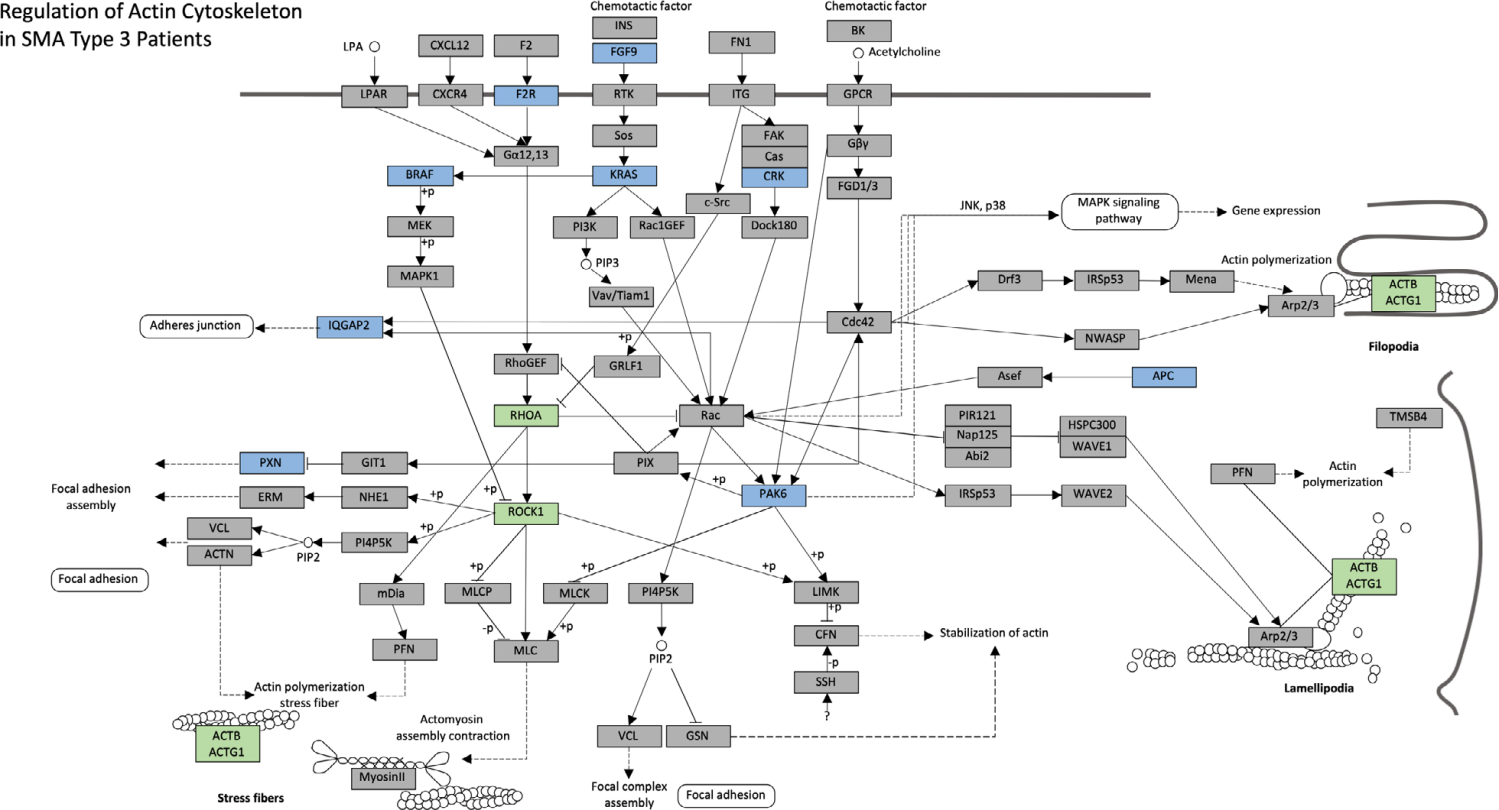
Regulation of Actin Cytoskeleton is significantly downregulated in SMA type 3 patients. KEGG pathway modified to highlight the genes in Regulation of Actin Cytoskeleton pathways that were significantly downregulated in the L1000 experiment (blue). Additional key genes in this pathway were highlighted (green) for further testing using RT‐qPCR. Of note, CRK, PAK6, PXN, and RHOA are included in the list of 978 "landmark" genes

We compared L1000 transcriptome profiles from SMA patients with age‐ and gender‐matched controls (see methods). With the goal of generating hypotheses about dysregulated expression pathways in SMA patients, we used a p‐value cutoff of *P* < 0.05. We found 270 significantly downregulated genes and 287 significantly upregulated genes in SMA patients compared to healthy controls (Fig. 1A). GSEA using differentially expressed genes revealed seven downregulated (Fig. 1B) and three upregulated (Fig. 1C) KEGG pathways^19^ associated with the immune system were downregulated in SMA patients, including “MAPK Signaling”, “T Cell Receptor Signaling,” and “Natural Killer Cell Mediated Cytotoxicity”. However, “Regulation of Actin Cytoskeleton” was the most significantly downregulated pathway in our analysis (Fig. 1B). We selected key genes from the core of this KEGG pathway to study further, including the small GTPase Ras homolog gene family member A (*RHOA*), the Rho‐associated protein kinase (*ROCK*), and genes that encode actin (*i.e., ACTB*, *ACTA1*, *ACTA2*, *ACTC1,* and *ACTG1*) (Fig. 2).

**Figure 3:**
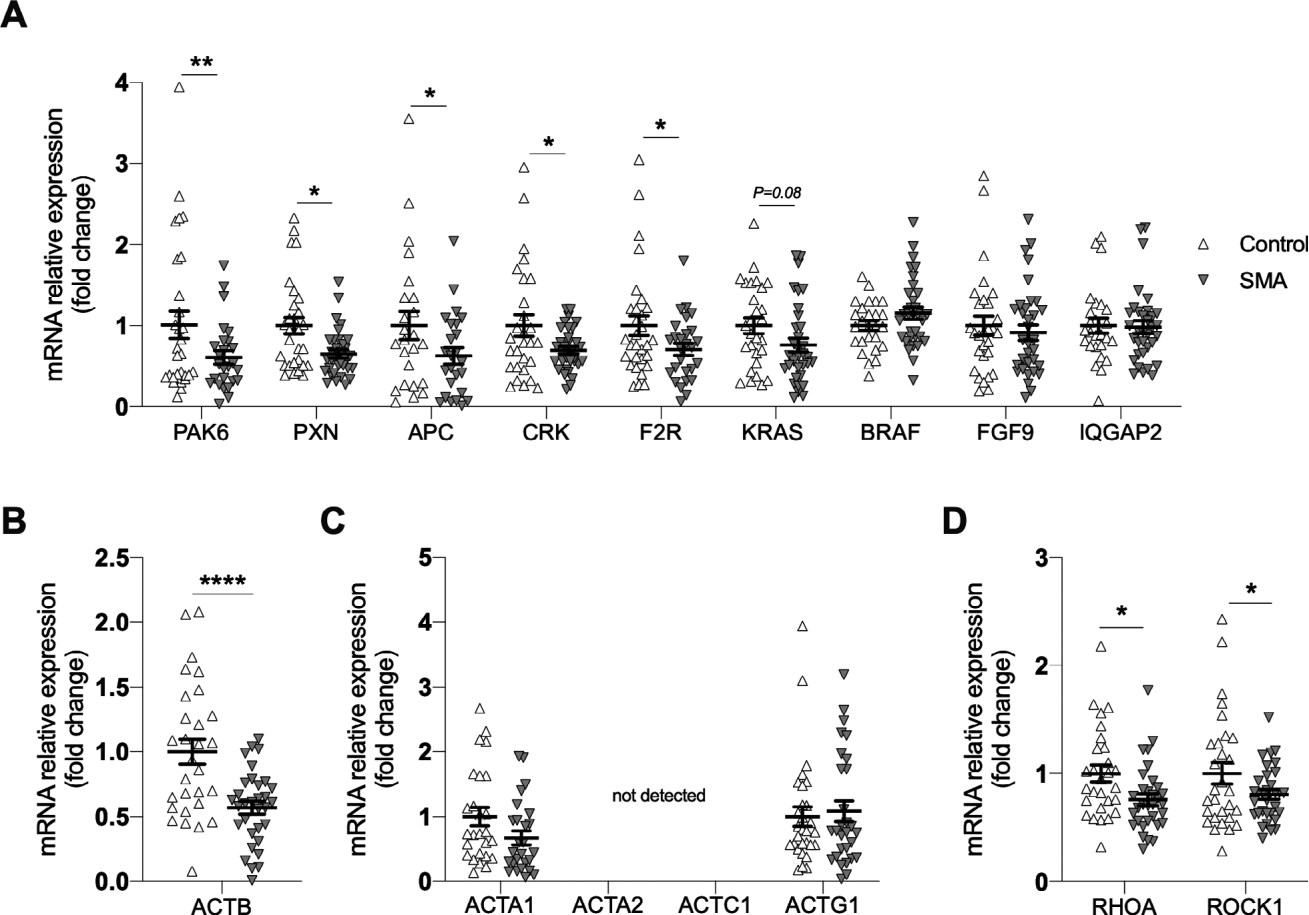
RT‐qPCR analysis of genes involved in Regulation of Actin Cytoskeleton in SMA type 3 patients (n = 31) and healthy controls (n = 34). (A) *PAK6, PXN, APC, CRK, F2R, KRAS, BRAF, FGF9, IQGAP2,* (B) *ACTB*, (C) *ACTA1*, *ACTA2*, *ACTC1*, *ACTG1*, and (D) *RHOA* and *ROCK1* mRNA expression. Unpaired Student’s *t* tests were used to compare groups. *P ≤ 0.05; **P ≤ 0.01; ****P ≤ 0.0001

RT‐qPCR analysis confirmed decreased expression of most of the genes identified in the initial L1000 analysis (Fig. 3A). SMA type 3 patients had a 43% decrease in *ACTB* mRNA expression compared to healthy controls (Fig. 3B). Moreover, SMA type 3 patients had a 24% decrease in *RHOA* mRNA expression and a 20% decrease in *ROCK1* mRNA expression (Fig. 3C). There were no significant changes in *ACTA1* and *ACTG1* mRNA expression (Fig. 3B) and RT‐qPCR analysis did not detect *ACTA2* or *ACTC1* mRNA. Together, these data help validate that actin cytoskeleton dynamics are downregulated in whole blood samples from SMA type 3 patients.

**Figure 4:**
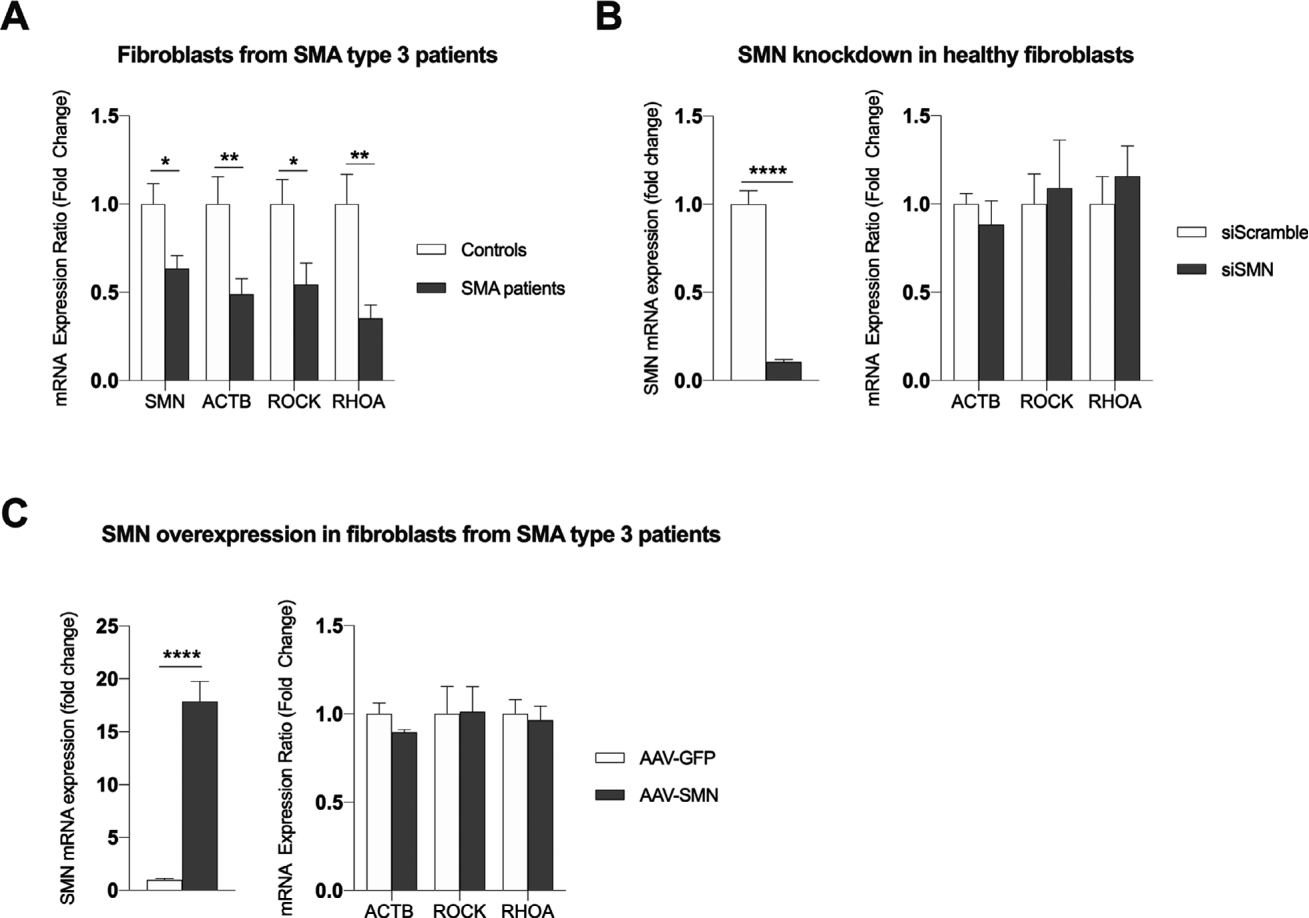
Regulation of actin cytoskeleton pathway in fibroblasts from SMA patients and family‐matched controls. (A) ACTB, RHOA, and ROCK1 mRNA expression in two independent cell lines with four technical replicates from independent experiments for each line. (B) Transient SMN knockdown in healthy fibroblasts using a complex of SMN siRNA and lipofectamine, n = 12 technical replicates. (C) *ACTB*, *RHOA,* and *ROCK1* mRNA expression in fibroblasts from SMA patients after SMN replacement using an AAV2 vector, n = 4 replicates from independent experiments. Unpaired (A) or paired (B and C) student’s *t* tests were used to compare groups. *P ≤ 0.05; **P ≤ 0.01; ****P ≤ 0.0001

To determine a potential cause–effect relationship between SMN levels and alteration of actin cytoskeleton dynamics, we performed experiments with fibroblasts derived from SMA type 3 patients and healthy controls. Fibroblasts derived from SMA type 3 patients had lower *ACTB, ROCK1,* and *RHOA* mRNA expression compared to fibroblasts derived from unaffected first‐degree relatives (Fig. 4A). However, transient SMN knockdown in fibroblasts from healthy subjects did not significantly affect *ACTB* mRNA expression (Fig. 4B), and SMN overexpression in fibroblasts derived from SMA patients did not affect *ROCK1*, *RHOA*, and *ACTB* mRNA expression (Fig. 4C). Altogether, these findings suggest that proteins other than SMN are contributing to the downregulation of actin cytoskeleton in SMA adult patients.

## Discussion

Understanding the contribution of systemic disease pathology in SMA could lead to new therapeutic avenues for our adult patients who have had limited access to FDA‐approved SMN‐modifying therapies. Here, we report data from the first cohort study that applied transcriptomic screening to peripheral whole blood samples from SMA type 3 patients and age‐ and gender‐matched controls as a method to identify significantly affected pathways. Our data demonstrate decreased expression of key genes involved in the regulation of the actin cytoskeleton in adult SMA patients. Furthermore, fibroblasts from SMA patients had reduced expression of key genes involved in the regulation of actin cytoskeleton, and SMN overexpression using an AAV vector in fibroblasts did not normalize the expression of these genes to the healthy control levels. Altogether, these data suggest that changes in the expression of key regulators of actin cytoskeleton are not directly associated with acutely reduced SMN levels. We speculate that changes in the regulation of actin cytoskeleton are therefore consequences of chronic low SMN levels, and acute overexpression is not sufficient to induce all adaptations necessary to reestablish the regulation of actin cytoskeleton. Importantly, while the impact of impaired actin cytoskeleton dynamics has been previously demonstrated in motoneurons from SMA type 1 patients,^12^, ^13^ these novel findings implicate altered actin cytoskeleton dynamics in peripheral cells and tissues in adult SMA patients.

Our data demonstrate decreased mRNA expression of genes encoding actin in the whole blood of adult SMA patients. The human genome contains six actin genes: *ACTA1*, *ACTA2*, *ACTB*, *ACTC*, *ACTG1*, and *ACTG2*. *ACTB* and *ACTG1* are expressed in all cells, while the other genes are mainly expressed in cardiac (*ACTC*), smooth (*ACTA2*), enteric (*ACTG2*), and skeletal muscles (*ACTA1*). *ACTB* was the only significantly downregulated among these six actin genes in the whole blood of SMA patients, indicating its potential importance as a biomarker of disease activity in SMA patients with chronic denervation. Further research is needed to determine whether the *ACTB* mRNA expression is altered in other cells and tissues, since these data were limited to SMA patient‐derived whole blood and fibroblasts.

The actin cytoskeleton is the primary force‐generating machinery in the cell. Actin filaments are uniformly oriented, and actin polymerization produces pushing forces which play an essential role in whole‐cell migration (for details, please see 10). This process is controlled by regulatory proteins, including the transforming protein RhoA, encoded by the gene *RHOA*, and the Rho‐associated protein kinase 1, encoded by the gene *ROCK1*. RhoA is a small GTPase that acts upon the effector of the kinase encoded by *ROCK1* to regulate actin stress fibers formation.^18^, ^21^ Diseases associated with *RHOA* mutations include adenocarcinoma^22^ and peripheral T‐cell lymphoma.^23^ Here, we found that *RHOA* and *ROCK1* mRNA expression, like *ACTB* mRNA expression, was downregulated in SMA patients. These findings indicate that other regulatory proteins from the actin cytoskeleton are downregulated in adult SMA patients, fostering enthusiasm that this pathway could include potential therapeutic targets to counteract SMA manifestations in tissues other than motor neurons.

The mechanisms underlying the observed downregulation of actin cytoskeleton dynamics is unknown. To our knowledge, direct interaction between SMN and actin has not been previously reported. However, SMN is known to interact with Profilin‐2^12^ and in the absence of SMN, Profilin‐2 interacts with actin monomers to result in actin polymerization.^24^ Moreover, reduced SMN levels increase the interaction between Profilin‐2 and ROCK.^25^ Interestingly, SMN knockdown in neuronal cell lines resulted in more filamentous actin (F‐actin) relative to globular actin (G‐actin).^13^ While we report mRNA expression data from whole blood and fibroblasts from SMA type 3 patients and controls in this study, future studies are necessary to determine the levels and phosphorylation state of these key proteins in the regulation of the actin cytoskeleton pathway in selected tissues in SMA patients.

One important clinical possibility raised by the current study is whether readouts related to alternations in the actin cytoskeleton pathway could serve as biomarkers to track SMA severity and disease progression. Identifying prognostic, predictive, and treatment responsive biomarkers to track disease status in SMA patients remains a significant challenge. Previous efforts to identify novel biomarkers for SMA include the BforSMA study. This cross‐sectional study applied an unbiased approach to screen plasma from a cohort of infantile and childhood‐onset SMA type 1, 2, and 3 patients and age‐ and gender‐matched controls to identify 97 proteins and 59 metabolites that correlated with SMA phenotypes across a wide spectrum of disease severity.^26^ Other exploratory biomarkers include immunoassays to detect circulating levels of light‐ or heavy‐chain neurofilaments, a marker of axonal damage,^27^ and serum creatinine, which we recently demonstrated to be a simple biomarker of progressive denervation in SMA.^28^ No studies to date have examined these potential biomarkers specifically in adult SMA patients. Additional biomarkers to better assess acute and chronic denervation, whole body lean mass, metabolic status, and other measures of health and well‐being in adult SMA patients are also needed. Readouts related to alterations in the actin cytoskeleton pathway should be considered.

We acknowledge our study has limitations. First, due to the large number of genes (10,174) reported using the L1000, many of the differentially expressed genes at *P* < 0.05 might be false positives. However, we believe there is value in using L1000 expression profiling with less stringent cutoffs in the hypothesis generation setting. With this direction, we have validated candidate genes and pathways with RT‐qPCR. An additional limitation is the limited sample size available for rare genetic diseases. Here, we could include a total of 34 SMA patients and 31 healthy control subjects. However, future studies including higher sample size and longitudinal cohorts would be valued to confirm or not our current findings. Lastly, we focused on the validation of changes in genes related to the regulation of actin cytoskeleton, which was the most significant downregulated pathway in SMA subjects. Potential changes in pathways related to immunoregulation and also pathways upregulated including multiple genes related to mitochondrial dysfunction are also interesting findings in this cohort study and future studies will be necessary to validate potential changes in specific immune cells in SMA patients.

In summary, these novel findings demonstrate downregulation of actin cytoskeleton in whole blood samples from SMA patients and provide new insights into the contribution of SMN deficiency in tissues other than motor neurons and therapeutic targets for SMA adult patients.

## Conflict of Interest

JJS, FCN, CRRA, BAS, NJL, EE, RG, SDSDL, AJJ, and AS report no disclosures. FCN is currently an employee of Biogen. KJS serves on the Cure SMA scientific advisory board, is a consultant for Biogen, Roche, and AveXis, and receives grant funding from Biogen. She is a principal investigator for the NURTURE (Biogen) and SPR1NT (AveXis) clinical trials.

## Supporting information


**Table S1.** Differentially regulated genes between SMA type 3 patients and healthy controls.
